# Enhancing Free Cyanide Photocatalytic Oxidation by rGO/TiO_2_ P25 Composites

**DOI:** 10.3390/ma15155284

**Published:** 2022-07-30

**Authors:** Elim Albiter, Jose M. Barrera-Andrade, Lina A. Calzada, Jesús García-Valdés, Miguel A. Valenzuela, Elizabeth Rojas-García

**Affiliations:** 1 Laboratorio de Catálisis y Materiales, ESIQIE-Instituto Politécnico Nacional, Zacatenco, Ciudad de México 07738, Mexico; ealbitere@ipn.mx (E.A.); jmanban@yahoo.com.mx (J.M.B.-A.); lina_calzada@yahoo.com.mx (L.A.C.); mavalenz@ipn.mx (M.A.V.); 2 Departamento de Química Analítica, Facultad de Química, Universidad Nacional Autónoma de México (UNAM), Ciudad Universitaria, Ciudad de México 04510, Mexico; jesusgv@unam.mx; 3 Área de Ingeniería Química, Universidad Autónoma Metropolitana-Iztapalapa, Av. San Rafael Atlixco 186, Col. Vicentina, Iztapalapa, Ciudad de México 09340, Mexico

**Keywords:** free cyanide, photocatalytic oxidation, Au–cyanide complex, graphene-TiO_2_ composites, Raman, FTIR, photoluminescence

## Abstract

Graphene-TiO_2_ composites have been investigated in various photocatalytic reactions showing successful synergy compared to pristine TiO_2_. In the present work, graphene oxide (GO) was synthesized by the Hummers method and then reduced graphene oxide-TiO_2_ composites (rGO/TiO_2_) were obtained by an in situ GO photoreduction route. X-ray diffraction, FTIR, Raman, UV–vis DRS, and photoluminescence were the main characterization techniques. The obtained composites containing 1 and 3 wt.% rGO were evaluated in the cyanide (50 mg/L) oxidation and Au-cyanide complex (300 mg/L) degradation under UV-A light. The composites showed higher photocatalytic activity than TiO_2_, mainly with the 1% rGO content. Cyanate and gold nanoparticles, deposited on the photocatalyst’s surface, were the main byproducts during the photocatalyst assessment. The improved photocatalytic activity of the composites was attributed to a higher rate of electron transfer and a lower rate of charge recombination due to the chemical interaction of rGO with TiO_2_.

## 1. Introduction

Anionic pollutants, (e.g., nitrate, cyanide, phosphate, fluoride, chromate, arsenate, and vanadate, among others) in water and wastewater have been investigated for decades due to their harmful effect on human health and ecosystems [1]. In particular, cyanides comprise compounds containing the −C≡N group, (e.g., HCN, KCN, K_4_[(Fe(CN)_6_], CH_3_CN), which are used in several industries such as mining, food processing, electroplating, coal coking, among others [2]. Free cyanide refers to the cyanide anion (CN^−^) and the HCN molecule, which coexist in equilibrium in the aqueous phase, depending on pH. For instance, at a pH less than 9.3, most of the total cyanide will exist as HCN molecules, while only the cyanide anion at higher pH will be present (T = 25 °C) [3]. Cyanide ions are commonly found in wastewater in a concentration ranging from 1–500 mg/L, depending on the industry that comes from, which is the most critical that of gold cyanidation solution (>500 mg/L) [4].

Free cyanide toxicity by inhalation, ingestion, or skin contact is well-known in human beings. It works by inhibiting the cytochrome oxidase enzyme, disrupting the process of cellular respiration [3,4,5]. Concerning the harmful effects of cyanides on living beings, different strategies have been used to eliminate free cyanides by their total conversion into harmless compounds such as carbon dioxide and nitrogen (2CN^−^ + 2O_2_ → 2CO_2_ + N_2_).

It is common practice to use chemical processes, (e.g., treatment with sulfur dioxide, chlorine, ozone), physical, (e.g., aeration, reverse osmosis), and biological, (e.g., enzymatic degradation), which have advantages and disadvantages in their operation [6,7,8]. On the other hand, heterogeneous photocatalysis represents a viable green alternative for cyanide degradation in wastewater, which is investigated using a wide variety of catalytic materials under different operating conditions [9,10].

Titanium oxide-based nanomaterials efficiently degrade and mineralize toxic organic and inorganic compounds in wastewater [11]. However, to improve its photocatalytic performance, in terms of the visible light absorption and suppression of charge carriers’ recombination, the modification of TiO_2_ with carbon materials has shown surprising results [12,13,14,15,16]. Graphene materials possess excellent properties, such as high specific surface area, outstanding charge carrier mobility, high thermal conductivity, high adsorption capacity/electrical conductivity, and high optical transparency. Moreover, they serve as an electron reservoir to accept and transport photogenerated electrons in the semiconductor, enhancing the adsorption capacity for reaction substrates and tuning the composites’ light absorption range and intensity [17,18,19]. As a result, the applications of graphene-based composites in photocatalysis, mainly in the remotion of organic pollutants, have exponentially grown, as observed by the number of publications in the last decade [20,21].

The photocatalytic degradation of free and metal cyanide complexes using graphene/TiO_2_ composites is scarce [22]. However, according to previous reports, graphene oxide showed promising applications as a photocatalyst and adsorbent to eliminate free cyanide and other harmful species [23,24]. Therefore, this current study aims to evaluate the photocatalytic oxidation of free cyanide and gold–cyanide by using rGO/TiO_2_ composites.

This research aims to increase the photocatalytic activity of TiO_2_ by adding reduced graphene oxide (rGO), improving the free cyanide degradation, and removing cyanide and metal simultaneously in a gold–cyanide complex. In this investigation, the effect of adding different amounts of rGO (1 and 3 wt.%) on the interaction with TiO_2_ and its role in photocatalytic degradation of free-cyanide and gold–cyanide complex were analyzed. The primary characterization techniques used to support these results were XRD, TEM, Raman, FTIR, photoluminescence, and UV–Vis spectroscopies.

## 2. Materials and Methods

### 2.1. Materials

Sulfuric acid (purity > 98%), potassium permanganate, and sodium nitrate (purity > 99.4%) were acquired from J.T.Baker; hydrogen peroxide (30% *v*/*v*), potassium hydroxide, potassium cyanide (KCN, purity > 97%), and potassium dicyanoaurate (I) (KAu(CN)_2_) from Sigma-Aldrich, ethylenediaminetetraacetic acid (EDTA) from Alyt brand, isopropyl alcohol, titanium dioxide (P25) from Evonik; tri-distilled mercury from Merck, and industrial-grade graphite. All reagents were used without further purification.

### 2.2. Preparation of rGO/TiO_2_ Composites

Firstly, graphene oxide was prepared using the Hummers method [25], and then reduced graphene oxide TiO_2_ composites (rGO/TiO_2_) were obtained by an in situ photoreduction method. Briefly, 2 g of TiO_2_ was added to 100 mL of anhydrous ethanol, and the desired amount of graphene oxide (GO) was included in the suspension to obtain 1 and 3 wt.% GO. The suspension was homogenized in an ultrasound bath (10 min) and then degassed for 5 min in a nitrogen atmosphere. The GO photoreduction was carried out in an UV-light photoreactor (LuzChem, Ottawa, ON, Canada, six low-pressure Hg lamps, λ_max_ = 365 nm) for 12 h at room temperature (25 °C) under vigorous stirring. Finally, the obtained solids were washed with deionized water and dried at 100 °C for 3 h.

### 2.3. Characterization

The DR UV–vis spectra were obtained using an Agilent Cary 100 spectrometer (Agilent Technologies, CA, USA) equipped with an integrating sphere. FTIR analyses were carried out on a NEXUS spectrometer (Thermo Scientific, Waltham, MA, USA) using the transmission KBr pellet technique in the range of 4000 to 400 cm^−1^. Raman spectra were recorded on an InVia Renishaw system (Renishaw plc., Gloucestershire, UK) equipped with a cooled CCD detector (−73 °C) and a holographic super-Notch filter. The samples were excited with the 532 nm Ar line; the spectral resolution was ca. 4 cm^−1,^ and spectrum acquisition consisted of 5 accumulations of 10 s. The photoluminescence (PL) spectra were obtained in an FLS1000 spectrophotometer (Edinburgh Instruments, Livingston, UK) using an excitation wavelength of 307 nm. The spectra were collected at room temperature with a spectral resolution of 0.5 nm and consisted of 5 accumulations of 0.1 s at each point. The catalyst morphology and particle size analyses were carried out using a scanning electron microscope model JSM6701F (JEOL). Powder samples were analyzed using the secondary electron mode. The textural properties of the catalysts were determined by N_2_ physisorption measurements at low pressure and 77 K, using an equipment model Autosorb iQ (Quanthachrome, VA, USA). The surface area was calculated using the BET model at 0.05 < P/P_0_ < 0.3 values of the adsorption isotherms. Moreover, the BJH model was used to estimate the pore size and volume from the desorption isotherms data.

### 2.4. Photocatalytic Evaluation

The photocatalytic degradation of free cyanide (CN^−^) and the gold–cyanide complex was carried out in a 100 mL glass cell under UV light irradiation (LuzChem photoreactor, six lamps, λ_max_ = 365 nm). In a typical experiment, 50 mg of catalyst were suspended, using an ultrasonic bath, in 50 mL of a KCN solution (50 ppm, pH = 12). After, a 400 mL/min airflow was injected into the glass cell space in total darkness under constant magnetic stirring for 30 min (adsorption–desorption process), and then the UV lamps were turned on. During the experiment, aliquots were taken every 30 min and filtered using a 0.2 μm filter (Millipore) to remove the catalyst. CN^−^ and cyanate ions were quantified in each sample using the differential pulse polarography (DPP) technique (797 VA computrace, Metrohm).

In the case of the gold–cyanide complex degradation experiments, an aqueous solution of KAu(CN)_2_ (300 ppm) and 500 µL of isopropyl alcohol as a sacrificial agent, and the experimentation followed the same protocol used for the degradation of free cyanides. The DPP technique was also used to analyze the [Au(CN)_2_]^−^ ion concentration, and it is the first time this technique has been reported in monitoring the Au–CN complex. Detailed information on the DPP technique analysis of free cyanide and cyanate is in the Supplementary Information (SI).

## 3. Results and Discussion

### 3.1. Characterization

**Figure 1:**
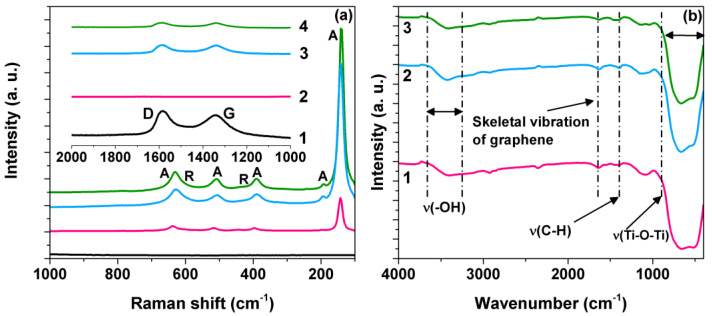
(**a**) Raman spectra of (1) graphene oxide, (2) TiO_2_ P25 (3) 1.0rGO/ TiO_2_, and (4) 3.0rGO/TiO_2_. (**b**) FTIR spectra of (1) TiO_2_ P25, (2) 1.0rGO/TiO_2_, and (3) 3.0rGO/TiO_2_. A: anatase, R: rutile.

The coexistence of rGO and TiO_2_ in the composite materials was detected by Raman spectroscopy. Figure 1a shows the Raman spectra of the TiO_2_, GO, and composite materials. In the TiO_2_ Raman spectrum, several bands are observed around 144, 396, 517, and 638 cm^−1^ characteristics of the E_g_, B_1g_, A_1g_ + B_1g,_ and E_g_ vibrational modes of anatase phase, respectively [26]. At the same time, two bands at 445 and 605 cm^−1^ were detected and attributed to the rutile phase’s E_g_ and A_1g_ modes. Graphene oxide (GO) has two characteristic signals at 1350 and 1600 cm^−1^ corresponding to the D and G bands. The D band is associated with sp^3^ defects within the hexagonal graphitic structure. In contrast, the G band is characteristic of sp^2^ hybridized carbon materials, providing information on the plane vibration of sp^2^ bonded carbon domains [27]. The D and G band ratio (I_D_/I_G_) is a parameter that indicates the disorder degree in the graphitic structure or edge. The I_D_/I_G_ ratio calculated for the materials were 1.56, 0.99, 1.04, and 1.36 for GO, rGO, 1.0 rGO/TiO_2_ and 3.0 rGO/TiO_2_. As can be seen, the 1.0 rGO/TiO_2_ composite shows the lowest I_D_/I_G_ ratio, smaller than that of GO, indicating an interaction between GO and TiO_2_ through π–π stacking. This decrease reveals an increase in sp^2^ domains in the carbon atoms planes due to decreasing unsaturated defects atoms in the GO by eliminating oxygenated functional groups and thus transforming GO to rGO [28,29,30].

FT-IR spectra of the TiO_2_ and rGO/TiO_2_ composite materials are displayed in Figure 1b and the GO spectrum are illustrated in Figure S1 (SI). In the GO spectrum, a broadband center at 3410 cm^−1^ is observed, characteristic of stretching vibrations of the ^−^OH bond due to adsorbed water molecules on the surface. Furthermore, the GO absorption spectrum also presented bands around 1721, 1574, 1389, and 1047 cm^−1^, attributable to stretching vibrations of carbonyl/carboxyl groups (C=O) present in the edges of graphitic layers, skeletal vibration of the graphene, bending vibration of tertiary C-OH hydroxyl groups, and stretching vibrations of =C-H groups, respectively [30,31]. Figure 1b shows FTIR spectra of TiO_2_ and composite materials in which a broad absorption band appears around 3409 cm^−1^, characteristic of the O-H stretching vibration of ^−^OH groups or adsorbed water. Other bands are perceived around 1579 and 1383 cm^−1^, which are characteristic of skeletal vibration of the graphene and C-H vibration, respectively. Additionally, a broad absorption band between 400 and 1000 cm^−1^ is detected in the FTIR spectra of TiO_2_ and rGO/TiO_2_ corresponding to Ti-O-Ti vibrations.

**Figure 2:**
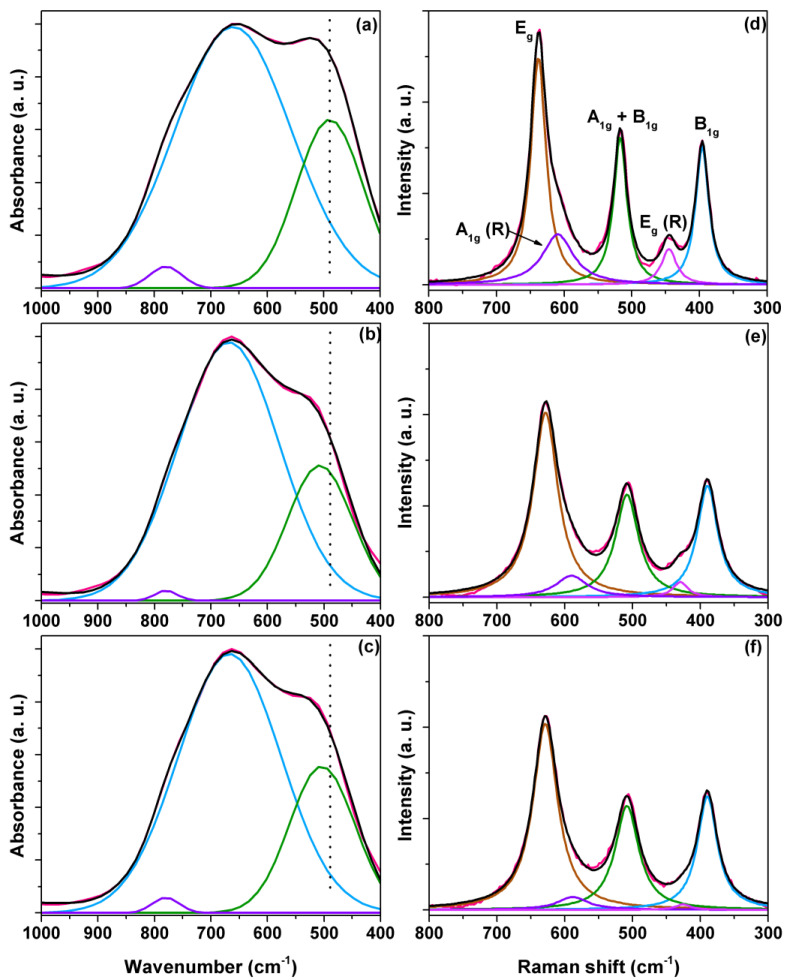
(**a**–**c**) Deconvolution of FTIR spectra of TiO_2_, 1.0rGO/TiO_2_, and 3.0rGO/TiO_2_, respectively. (**d**–**f**) Deconvolution of Raman spectra of TiO_2_ Evonik, 1.0rGO/TiO_2_, and 3.0rGO/TiO_2_, respectively. A: anatase, R: rutile.

However, it has been a point of debate in recent years to determine the band’s position corresponding to the interaction of TiO_2_ nanoparticles and rGO sheets via the possible formation of the Ti-O-C bond [32,33]. Thus, this zone was deconvoluted, and the results are displayed in Figure 2a–d. It can be noted that the broad absorption band between 400 and 1000 cm^−1^ for TiO_2_ and the composite materials show three components at 489, 660, and 783 cm^−1^, which have been assigned to Ti-O-Ti vibrations [34,35]. Furthermore, a displacement in the band position at 489 cm^−1^ to higher wavenumbers for 1.0rGO/TiO_2_ and 3.0rGO/TiO_2_ composites were observed, possibly due to a chemical interaction between TiO_2_ nanoparticles and rGO [30,36]. Interestingly, our FTIR results do not support the formation of a Ti-O-C bond since the deconvolution analysis in the 1000 cm^−1^ region always showed three components, even though the rGO is not present in the photocatalyst.

The presence of Ti-O-C bonds improves the separation of photogenerated electrons and holes after light irradiation, decreasing the charge carrier’s recombination and giving rise to a higher photocatalytic activity [31,34]. The deconvolution of the Raman spectra was performed in the range of 300 and 800 cm^−1^ for TiO_2_ and composite materials to support a Ti-O-C bond formation, as shown in Figure 2d–f. As can be seen, three components at 635 (E_g_), 516 (A_1g_ + B_1g_), and 396 (B_1g_) cm^−1^ correspond to the anatase phase, and two components at 608 (A_1g_) and 433 (E_g_) cm^−1^ belong to the rutile phase, in the deconvolution of the Raman spectra are observed. The intensity of these last two components decreases with the increment of the rGO content. Additionally, an increase in the full width at half maximum (FWHM) of the rutile A_1g_ and E_g_ Raman peaks in the composite materials was observed (Figure 2e). Shahbazi et al. [37] attributed the widening of the rutile E_g_ Raman peak to the distortion of the titania framework host due to the introduction of rGO into the titania lattice.

**Figure 3:**
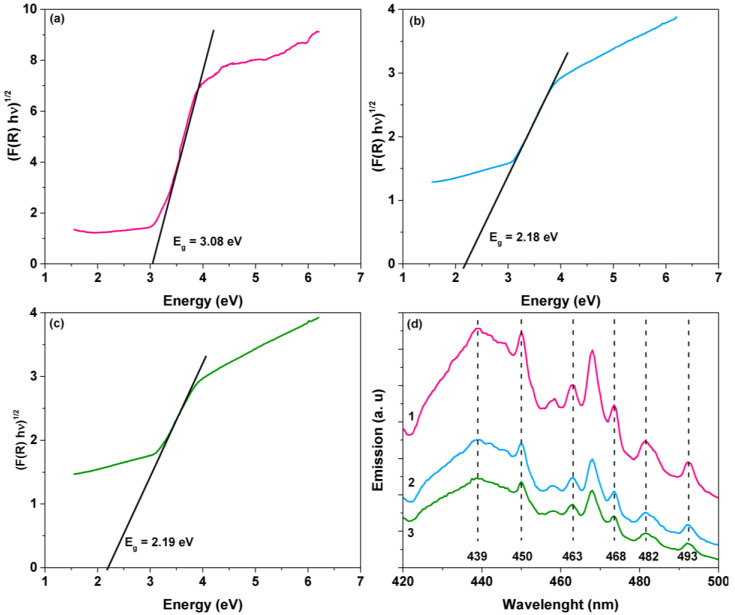
Tauc plots for the determination of optical band gap of (**a**) TiO_2_, (**b**) 1.0rGO/TiO_2_, and (**c**) 3.0rGO/TiO_2_, respectively. (**d**) PL spectra of (1) TiO_2_ P25, (2) 1.0rGO/TiO_2_, and (3) 3.0rGO/TiO_2_.

The photocatalysts were characterized using UV–Vis diffuse reflectance spectroscopy to know the effect of the amount of rGO in the composite materials on the optical properties. The composite materials’ optical bandgap was determined using the Tauc model [38], Figure 3a–c. The bandgap was obtained by plotting a tangent line to the point of inflection of the curve of the graph (αℎ𝜈)^1/2^ vs. ℎ𝜈 (energy), where (αℎ𝜈)^1/2^ = 0. A decrease (redshift) was observed, due to the incorporation of the rGO into the TiO_2_ P25 (3.08 eV). The resulting bandgap values for the composite materials were: 2.18 and 2.19 eV for 1% rGO, and 3% rGO, respectively. Other studies have reported the same behavior [30,34]. This decrease in the bandgap could be attributed to the chemical interaction between the TiO_2_ nanoparticles and rGO nanolayers, resulting in increased photocatalytic activity in the composites compared to pristine TiO_2_.

The photoluminescence spectra of TiO_2_ and rGO/TiO_2_ composites were determined at steady-state and room temperature under an excitation wavelength of 307 nm (Figure 3d) to verify the charge carriers’ recombination process. The presence of rGO in the composites causes a decrease in the PL intensity, depending on its loading. This behavior could be explained by suppressing the electron–hole recombination in the TiO_2_ [39]. Additionally, the peaks at PL spectra can be ascribed to several defects due to self-trapped excitons, oxygen vacancies, or other surface states [40]. For example, the peaks at 439 nm could be assigned to the self-trapped exciton caused by the de-excitation of Ti^3+^_3d_ electrons to the valence band [41]. The same can be said for the 450, 463, and 468 nm signals. Finally, the peaks at 482 and 493 nm could be caused by oxygen vacancies [41].

TiO_2_ (Figure S3a) indicates the presence of a nanometric powder made of dense and equiaxial particles exhibiting a particle diameter of ca. 30 nm. The analyses by scanning electron microscopy and nitrogen physisorption were performed to know the effect that the addition of rGO to TiO_2_ has on the bulk and surface properties, respectively. Figure S3 shows the SEM analysis of the samples. The morphology and observed primary particle size did not change significantly in the samples of 1.0rGO/TiO_2_ (Figure S3b) and 3.0rGO/TiO_2_ (Figure S3c). However, certain agglomerates were observed, attributed to the incorporation of the rGO phase, which the TiO_2_ must cover as the former is present in the sample as a minority component. Generally, the obtained samples are homogeneous, with no phase segregation observed.

Figure S4 shows the obtained N_2_ adsorption/desorption isotherms. Based on the UIPAC classification, TiO_2_ exhibits a type II isotherm characteristic of dense, non-porous solids. This result agrees with SEM observations. Moreover, the specific surface area values were calculated using the BET model and are presented in Table S1. As expected, incorporating rGO into the TiO_2_ promotes a higher specific surface area for the 3.0rGO sample. Some changes are observed in the isotherms profile, wherein the hysteresis loop area increases as the rGO increases; in the same sense, an increase in the pore volume is evidenced due to the incorporation of the rGO on TiO_2_ particles.

### 3.2. Photocatalytic Activity

Before the photocatalytic evaluation, three blank experiments were performed. First, no cyanide losses were observed by volatilization in the reaction medium at pH = 12 after three hours. Second, the cyanide conversion in the absence of a photocatalyst was negligible, even under UV light irradiation. Third, the cyanide reaction in the presence of the photocatalyst, but without UV light, did not proceed. Additionally, CN^−^ adsorption was performed on TiO_2_ and rGO/TiO_2_ composites; in these experiments, the CN^−^ concentration decreased ca. 10% after 60 min in the dark, under constant stirring. The only byproduct detected in the whole irradiation time was the cyanate ion.

**Figure 4:**
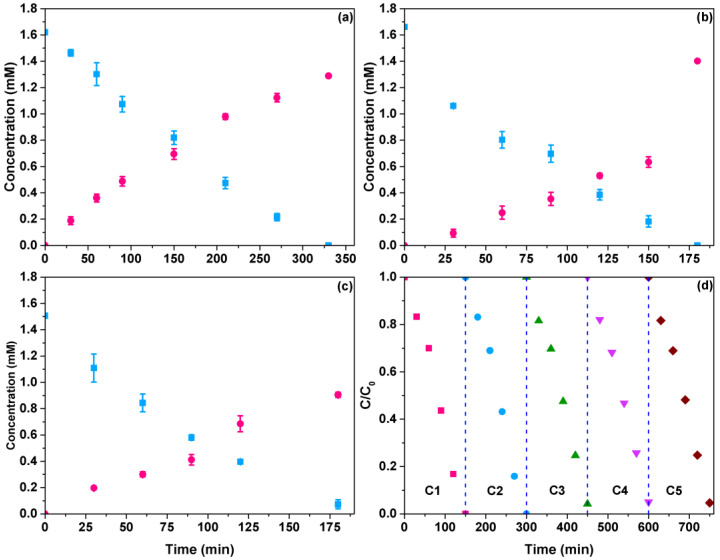
Degradation profiles of cyanide (■) and formation profiles of cyanate (●) using TiO_2_ and rGO/TiO_2_ composites: (**a**) TiO_2_, (**b**) 1.0rGO/TiO_2_, (**c**) 3.0rGO/TiO_2_. (**d**) The efficiency of cyanide photocatalytic oxidation in five cycles of the 1.0rGO/TiO_2_ composite.

**Figure 5:**
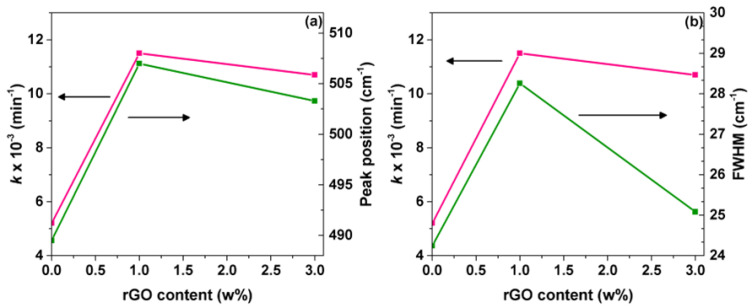
Influence of the composite’s rGO content on the reaction constant, (**a**) influence of the rGO content on the Ti-O-Ti stretching vibration peak position in the FTIR, (**b**) influence of the rGO content on the FWHM of the Rutile Eg Raman peak.

The degradation profiles of CN^−^ and cyanate ions (CNO^−^) using TiO_2_ and the rGO/TiO_2_ catalysts are presented in Figure 4. Interestingly, complete cyanide degradation was observed with all photocatalysts at different irradiation times, depending on the rGO concentration in the composites. For example, TiO_2_ required 335 min of irradiation to achieve the total cyanide conversion. Similar results were obtained by Kim et al. [42] but using 30 ppm of cyanide and irradiating with a UV-C lamp (180–280 nm). Note that the concentration profiles, (i.e., cyanide and cyanate) by using TiO_2_ (Figure 5a) or the rGO/TiO_2_ photocatalysts (Figure 5b,c) seem the same. Still, a faster reaction rate can be observed and corroborated by the shorter degradation time required (150–180 min). As a result, the presence of rGO improves the photocatalytic activity of TiO_2_ significantly, transforming CN^−^ into CNO^−^, a less toxic byproduct (CN^−^ + 2OH → CNO^−^ + H_2_O).

A photocatalyst’s stability and reusability are essential in determining catalytic materials’ practical utility. Thus, high stability is shown by the 1% rGO/TiO_2_ composite after five cycling tests are worth noting, which lost three percentage points after the third cycle and subsequently remained constant, indicating the composite’s robustness in the highly alkaline medium (Figure 4d).

Under our experimental conditions, cyanide degradation followed pseudo-first-order kinetics independently of the used photocatalyst. The calculated values, in min^−1^, of the apparent reaction rate constant (*k*) were 0.0052, 0.0115, and 0.0107 for TiO_2_, 1.0rGO/TiO_2_, and 3.0rGO/TiO_2_, respectively. According to these values, the best photocatalyst was 1.0 rGO/TiO_2_. This behavior has been previously observed in other rGO/TiO_2_ composites, where an excess of rGO decreased the photocatalytic performance of the materials [43,44]. This decrease is normally attributed to the higher rGO coverage of the TiO_2_ surface, which reduces the semiconductor’s light absorption.

Two routes are known to achieve the photocatalytic oxidation of cyanides in an aqueous solution at high pH (>10): indirect, which employs the photogenerated OH radicals in a homogeneous phase, and direct, by using the trapped holes at the surface hydroxyls groups [45]. Even though we have no evidence to support one or the other conversion pathway for cyanides, the presence of rGO improved the TiO_2_ photoactivity, which could be linked to a higher ROS (O_2_·, HO_2_·, HO·, among others) formation due to a reduced electron–hole pairs recombination rate [28]

As mentioned before, adding small amounts of rGO to TiO_2_ modifies its optical properties, confirming the chemical interaction between the two species. In other words, the observed slight changes in the TiO_2_ peak position (FTIR) and an increase in the FWHM of the Rutile Raman peak in the composite materials can be correlated with the rate constant for CN^−^ photocatalytic oxidation, as is shown in Figure 5.

**Figure 6:**
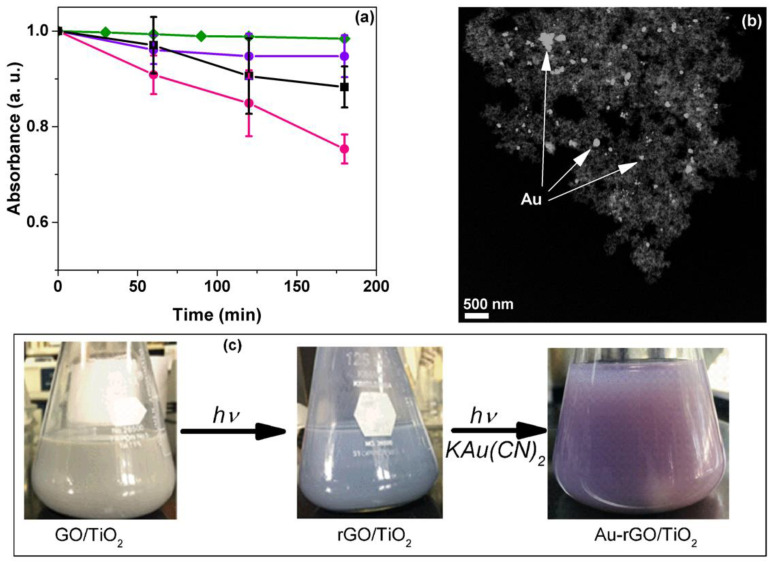
(**a**) Degradation profiles of Au(CN)_2_^−^ complex using rGO/TiO_2_ composites: (♦) photolysis, (●) TiO_2_ P25, (●) 1.0rGO/TiO_2_, and (■)3.0rGO/TiO_2_. (**b**) Dark field TEM image of the rGO/TiO_2_ composites after Au–N complex degradation. (**c**) Color changes during the synthesis of the rGO/TiO_2_ composite and degradation of the Au–CN complex.

This work introduces the results of the photocatalytic degradation of the Au–CN complex using rGO/TiO_2_ composites_._ The degradation profiles of the Au–CN complex (potassium dicyanoaurate at an initial concentration of 300 ppm) are shown in Figure 6a. The metal complex was degraded in the presence of isopropyl alcohol (10 mM) as a sacrificial electron donor and ambient air. Under these experimental conditions, the reduction in the gold–cyanide complex forms metallic gold and free cyanide according to the following reaction [46]: Au(CN)_2_^−^ + e^−^ → Au + 2 CN^−^.

Experimental evidence of the gold-cyanide degradation is presented in Figure 6b. A dark field TEM micrograph shows that gold particles appear deposited on the 1% rGO/TiO_2_ catalyst with different sizes. Note that the Au–CN complex degraded linearly with a clear dependence on the rGO amount. Surprisingly, the higher photocatalytic degradation (~30%) was obtained with the 1% rGO/TiO_2_, and the TiO_2_ presents a negligible conversion of the Au–CN complex. An increment in the rGO content in the composite materials (3% rGO/TiO_2_) and a decrease in photocatalytic activity were observed. This decrease could be due to the strong absorption of light or competition for light capture between reduced graphene oxide and TiO_2._ Figure 6c shows the various stages of the evolution of the graphene/TiO_2_ composites, from the integration of GO (sand color) to its transformation into rGO by photocatalytic reduction (gray color), and the deposit of gold particles on the composite (purple color). These results demonstrate an improvement of the photocatalytic activity of TiO_2_ again by adding rGO, which could explain a decrease in the recombination rate of electron–hole pairs, allowing the decomposition of a highly stable Au–CN complex.

**Figure 7:**
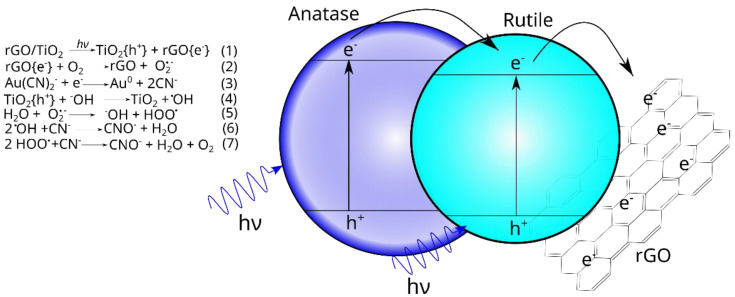
A schematic diagram showing the various steps for the Au–CN complex degradation in the presence of the rGO-TiO_2_ P25 composite under UV light.

A schematic proposal to explain the Au–CN complex photocatalytic reduction is presented in Figure 7. The first step consists of the Au(CN)_2_^−^ anion adsorption on the composite’s surface in an aqueous solution at pH 12 [47,48]. In the second step, UV light irradiation starts with the generation of the electron–hole pairs and promotes the Au(CN)_2_^−^ reduction and isopropyl alcohol oxidation, respectively. Experimental evidence of these reactions was the metallic gold particles deposited on the composite and the initial generation of acetone from isopropyl alcohol oxidation. In the third step, the deposited Au particles enhanced the photocatalytic oxidation of formed cyanide ions (Equation (3)) to cyanate. The fourth stage corresponds to the reaction between surface hydroxyls linked to TiO_2_ and the photogenerated holes producing OH radicals (Equation (4)). The subsequent steps (Equations (5)–(7)) involve reactions of the other radicals formed with the cyanide for its transformation into cyanate.

## 4. Conclusions

The photocatalytic reduction of graphene oxide successfully obtained reduced graphene oxide/TiO_2_ composites. Both composites presented a higher photocatalytic activity on free cyanide oxidation than TiO_2_, but 1% rGO loaded to TiO_2_ showed the best performance. The enhanced activity was attributed to the interaction between the rGO and TiO_2_, as evidenced by Raman, FTIR and UV–Vis, and photoluminescence studies. This interaction would reduce the charge carrier’s recombination rate, leading to an effective activation of TiO_2_ by UV irradiation and an enhanced charge transfer between RGO and TiO_2_, increasing the photocatalytic activity measured as cyanide degradation. In addition, the optical properties of the composites revealed a chemical interaction (Ti-O-C) between the TiO_2_ and rGO, which was correlated with the rate constant in the photocatalytic oxidation of cyanide.

Additionally, the rGO/TiO_2_ composites showed higher activity than TiO_2_ in the degradation of the Au–CN complex at high initial concentrations (300 ppm). The Au^−^ ion was successfully reduced and deposited on the surface of TiO_2_, as evidenced by TEM analysis.

## Supplementary Materials

The following supporting information can be downloaded at: https://www.mdpi.com/article/10.3390/ma15155284/s1, it includes the method for free cyanide, cyanate, and gold–cyanide complex analysis by DPP, Figure S1: FTIR GO spectrum. Figure S2: Plots obtained from the free cyanide degradation, Figure S3: SEM images of TiO_2_ and rGO/TiO_2_ composites, Figure S4: Nitrogen physisorption isotherms of TiO_2_ and rGO/TiO_2_ composites, Table S1: Textural properties of the different catalysts.
