# Supplementary material for: Enhancing Free Cyanide Photocatalytic Oxidation by rGO/TiO2 P25 Composites
Source: Materials (Basel). 2022 Jul 30;15(15):5284. doi: 10.3390/ma15155284 (PMC9369652; doi:10.3390/ma15155284)
Supplement: Supplementary file 1 [file materials-15-05284-s001.zip › materials-1820623-supplementary.pdf]

Supplementary Information

Enhancing Free Cyanide Photocatalytic Oxidation by rGO/TiO_2 P25 Composites

Elim Albiter [1], Jose M. Barrera-Andrade [1], Lina A. Calzada [1], Jesús García-Valdés [2,†], Miguel A. Valenzuela [1] and Elizabeth Rojas-García [3,*]

[1] Laboratorio de Catálisis y Materiales, ESIQIE-Instituto Politécnico Nacional, Zacatenco, Ciudad de México 07738, Mexico; ealbitere@ipn.mx (E.A.); jmanban@yahoo.com.mx (J.M.B.-A.); lina_calzada@yahoo.com.mx (L.A.C.); mavalenz@ipn.mx (M.A.V.)

[2] Departamento de Química Analítica, Facultad de Química, Universidad Nacional Autónoma de México (UNAM), Ciudad Universitaria, Ciudad de México 04510, Mexico; jesusgv@unam.mx

[3] Área de Ingeniería Química, Universidad Autónoma Metropolitana- Iztapalapa, Av. San Rafael Atlixco 186, Col. Vicentina, Iztapalapa, Ciudad de México 09340, Mexico

* Correspondence: erg@xanum.uam.mx

† Diseased.

It includes the method for free cyanide, cyanate, and gold–cyanide complex analysis by DPP

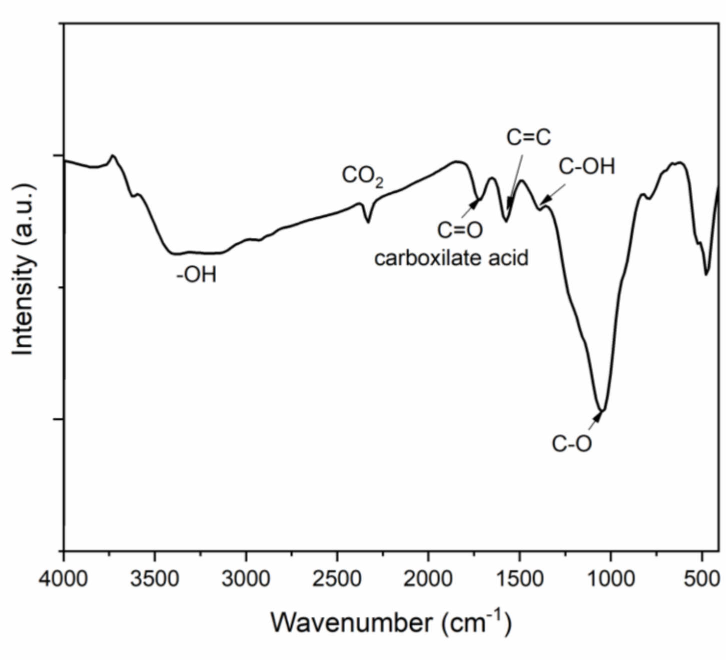

Figure S1. FTIR GO spectrum.

Free cyanide, cyanate, and gold-cyanide complex analysis by DPP

A potassium cyanide solution (50 ppm) was prepared, and the pH was carried to pH=12.0, where cyanide is almost 99% in the solution and thus avoids the formation of hydrocyanic acid. The pH of the deionized water was adjusted at 12.0 (before dissolving the potassium cyanide) with potassium hydroxide (KOH). 50 mL of the potassium cyanide solution was taken and placed into the reactor. Then, 50 mg of the catalyst or composite was added (to get 1 g/L), sonicated for 10 minutes, and the reactor was placed inside the LuzChem in total darkness and kept under constant magnetic stirring. The air was injected into the rector 400 mL/min for all the experiments. The air did not bubble up into the solution, and the bubbles cause stripping of the cyanide. For that, the air was put on the free space between the solution and the rubber stopper. See Fig. S2. 30 minutes for the cyanide adsorption-desorption on the composite surface, a sample was taken, and the UV lamps were turned on. The experiment was UV irradiated for more than 300 minutes, and samples were taken every 30 minutes for the first two hours after that sampling was performed every hour until the experiment finished. All the samples, to be analyzed, were filtered using a 0.2 μm filter (Millipore disk) to remove the catalyst. All experiments were done for triplicate and calculated the corresponding error bars.

In each sample, free cyanide and cyanate ions were quantified since they indicate the progress of the photocatalytic reaction. Free cyanide measurement was carried out using a differential pulse polarography (DPP).

The polarographic recordings were performed in an electrochemical cell with the next array of electrodes: working, mercury drop, counter electrode platinum wire, and the reference, Ag/AgCl/KCl. The polarograms were acquired using hardware and software from the same company (Methrom 797 VA Computrace). 99.99% pure nitrogen was bubbled through the solution to avoid oxygen interference. After each polarographic determination, the mercury was recovered and stored until having 50 mL. The mercury pollution was minimized by recycling. The mercury was treated and cleaned with NaOH, HNO3, and deionized water successively. This mercury was carefully stored and tested to confirm the absence of impurities and used in different determinations.

The supporting electrolyte for free cyanide polarographic determination was an H3BO3/KOH solution (0.1mol/L) prepared using deionized water. A free cyanide calibration curve was prepared. Increasing typical peaks were found when standards with higher concentrations were tested, see Fig. S3. The free cyanide determination was done in 10 mL supporting electrolyte; after adding 100 mL of the sample, and purging the cell with nitrogen for 300 seconds, the nitrogen was wetted with deionized water before entering the cell. The concentration of free cyanide was obtained using the differential pulse polarography (DPP) technique. The measurements of free cyanide were determined with 0.05 V pulse amplitude and 0.0099 V/s sweep rate. All the determinations were done for triplication.

For the determination of cyanate, the methodology of 2-amino benzoic acid (anthranilic acid) was followed, which consists of taking 0.5 mL of sample and placing it in a test tube, 0.5 mL of 0.01M solution of anthranilic acid was added, stirred, and then put into the oven at 40 °C for 10 minutes. Once it is cold, 1.0 mL of HCl 6N solution was added and stirred again. After that, the test tube was placed into boiling water for one minute to complete the reaction. The cyanate reacted with anthranilic acid to get a complex and quantified at 310 nm by using a calibration curve.

The tests with gold-cyanide complex were obtained as follows: a solution of potassium dicyanoaurate at a concentration of 300 ppm was prepared, the pH value of it was 12 because the cyanide is almost 99% in the solution and thus avoiding the formation of hydrocyanic acid. The pH was adjusted with potassium hydroxide 0.01 M (KOH), 50 mL of the potassium dicyanoaurate solution was taken and placed in a reactor. Then, 50 mg of the composite was added to have a concentration of 1 g/L. Then, it was sonicated for 10 minutes and placed inside the LuzChem in total darkness, and it was kept under constant magnetic stirring. It was left for 30 minutes for the adsorption-desorption process of the complex on the surface of the composite. At the end of this time a sample was taken, and the UV lamps were turned on and, the isopropanol was added to the reactor (500 μL). The experiment was left under UV irradiation for 180 minutes, samples were taken every 60 minutes until the experiment was completed. The samples, before any analysis, were filtered using a 0.2 μm Millipore disk to remove the catalyst. All experiments were done for triplicate and the corresponding error bars were placed.

The measurement of gold (Au (I)) was carried out using a polarographic method. This consisted of using the 797 VA computrace equipment of the Metrohm brand with an array of electrodes: working electrode (mercury drop), auxiliary electrode (platinum), and the standard Ag/AgCl electrode.

A KOH/EDTA solution with a concentration of 0.1M was used as a supporting electrolyte for the gold (Au (I)) determination, this electrolyte was prepared using deionized water. Au (I) calibration curve was previously made using the 797 VA computrace equipment and Au standard solutions for different concentrations. The procedure to measure the Au (I) concentration consisted: first putting 10 mL of the supporting electrolyte in the cell of 797 VA equipment, then 500μL of every sample (Au standard solutions) was put on, the cell was purged injected nitrogen for 300 seconds and finally, the calibration curve was made it. The concentration Au (I) was measured using the same procedure and finally quantify by utilizing the calibration curve.

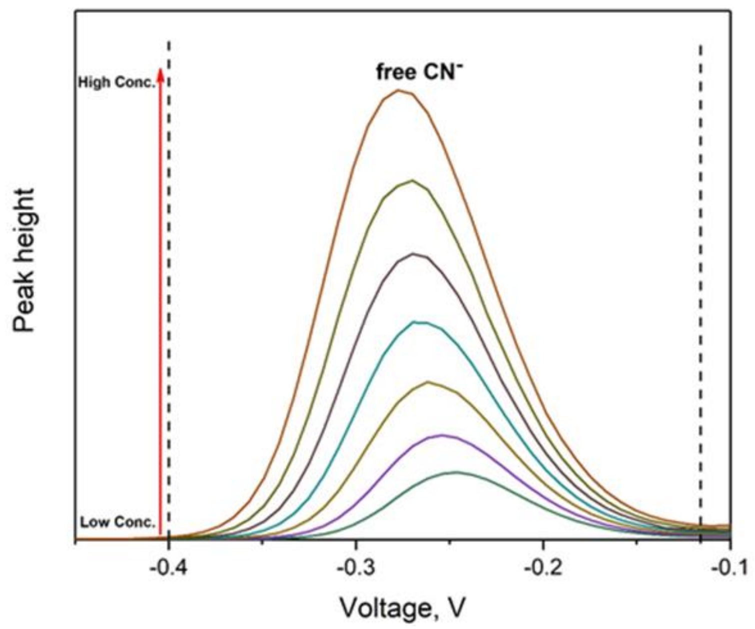

Figure S2. Plots obtained from the free cyanide degradation.

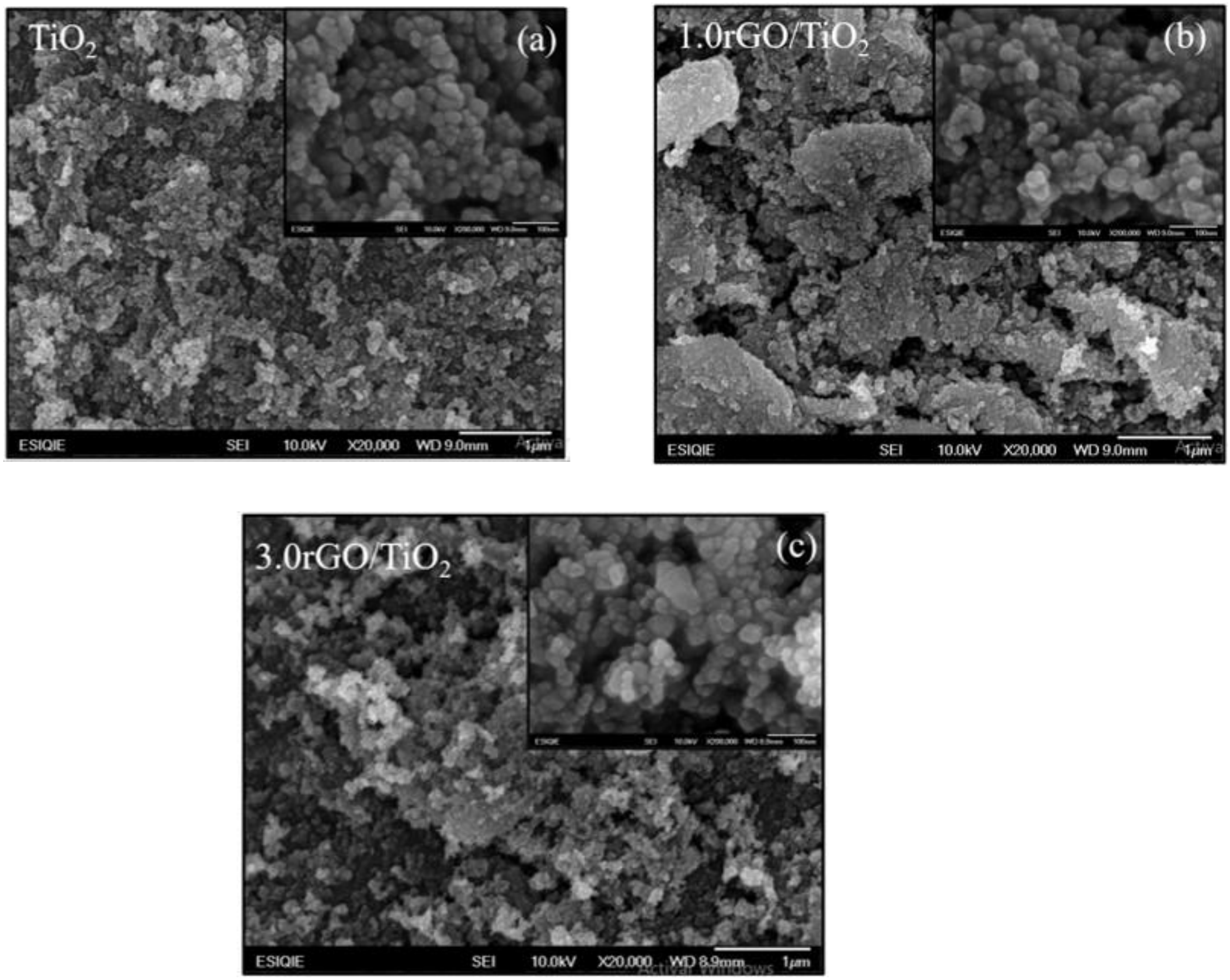

Figure S3. SEM images of TiO_2 and rGO/TiO_2 composites

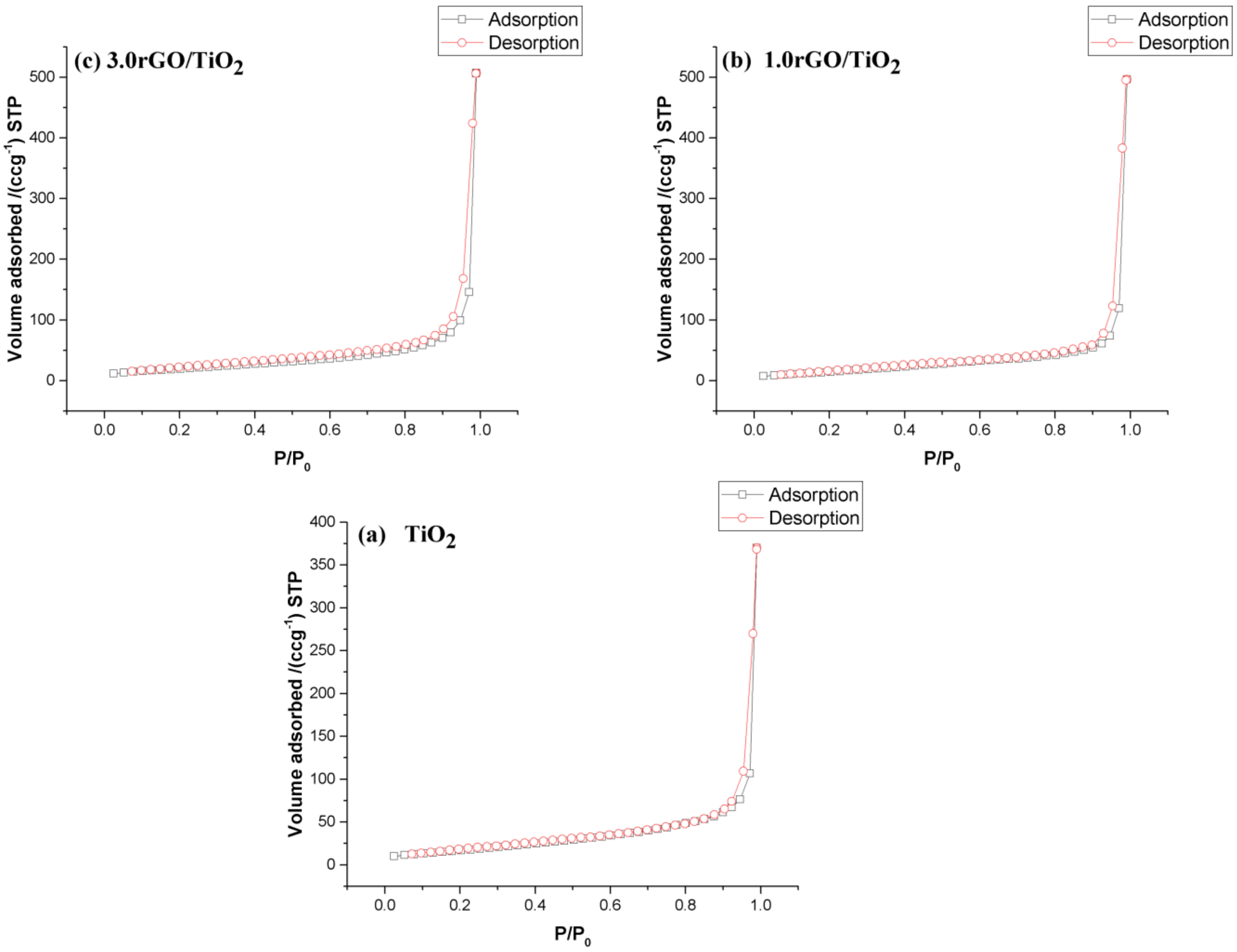

Figure S4. Nitrogen physisorption isotherms of TiO_2 and rGO/TiO_2 composites

Table S1. Textural properties of the different catalysts.

Sample	SSA_{BET} (m^2/g)	Mean pore diameter[a] (nm)	Total pore volume[b] (cm^3/g)
TiO_2	64.8	3.11	0.56
1.0rGO/TiO_2	60.4	3.11	0.76
3.0rGO/TiO_2	73.5	3.12	0.77

a Calculated based on desorption isotherms and BJH model.

b Calculated based on N2 adsorption and P/P_0 =0.975.
